# The complete mitogenome of *Chlorophanus auripes* Faust, 1897 (Coleoptera, Curculionidae)

**DOI:** 10.1080/23802359.2022.2086495

**Published:** 2022-06-20

**Authors:** Xiaoning Zhang, Guoxiang Zhang, Qingbai Hou

**Affiliations:** a College of Life Sciences, Qinghai Normal University, Xining, PR China; b Academy of Plateau Science and Sustainability, Qinghai Normal University, Xining, PR China

**Keywords:** Mitogenome, Weevils, Curculionidae, *Chlorophanus auripes*

## Abstract

*Chlorophanus auripes* Faust, 1897 is a large size weevil dwell in biotopes on *Salix spp*. or *Populus spp.*, which can be found common in Northern China. The complete mitogenome of *C. auripes* was reported in the present study. The mitogenome contains 18,149 base pairs. The composition of mitogenome is 39.4% for A, 10.1% for G, 36.0% for T and 14.5% for C. A set of 37 genes, including 13 protein-coding genes (PCGs), 22 tRNA genes, 2 rRNA genes and one control region, were annotated. There is a 1037 bps gap between Ile tRNA gene and Gln tRNA gene. The phylogenetic result supports the monophyly of Curculionidae, revealed that *C. auripes* was a sister group to the *Pachyrhinus yasumatsui*, which also belongs to the subfamily Entiminae.

## Introduction

*Chlorophanus* is a large weevil genus contains nearly 100 species, which belongs to the subtribe Tanymecina Lacordaire, 1863 of tribe Tanymecini Lacordaire, 1863 in subfamily Entiminae Schoenherr, 1823. They are large size (6.9–14 mm) and with a body being usually covered with green scales (Legalov 1997). The *C. auripes* is distributed in Northern China, Japan, and Korea (Han 2014). They dwell in biotopes on *Salix spp.* Or *Populus spp.* (Reitter 1915).

## Materials and methods

The adults *C. auripes* samples were collected in August 2020 from Xining, China (N36°40′45″, E101°44′50″). The specimens were deposited into absolute ethanol and stored under －20 °C in the Insect Collection of Qinghai Normal University, Xining, China (Qingbai Hou, bleding@126.com) under the voucher number QNU2020C000051. The total genomic DNA was extracted from the thorax muscles of a single individual using the TIANGEN Genomic DNA Extraction Kit (TIANGEN, Beijing, China) according to the manufacturer’s instructions. A total of four pairs overlapped PCR primers (Pair 1: TM-J211: 5′-AAGCTANTGGGTTCATACCCC-3′, N3-N5730: 5′-TTAGGRTC AAATCCRCATTC-3′; Pair 2: C3-J5578: 5′-TTGACTTCCAATCAAAARATCT-3′, CB-N11337: 5′-AAATATCATTCWGGTTGGATATG-3′; Pair 3: CB-J10942: 5′-TGAGGNCAAATATCMTT YTGAGG-3′, SR-N14613: 5′-CTAGGATTAGATACCCTATTAT-3′; Pair 4: LR-J12887: 5′-CCGGTTTAAACTCAGATCATGTAA-3′, C1-N2352: 5′-GCTCGTGTRTCTACATCTATHCC-3′) were designed to amplify the complete mitochondrial genome of *C. auripes*. The KOD-Plus (http://www.bio-toyobo.cn/product_detail_13.html) were used for amplification and the procedures were listed as following: the initial denaturation step was performed at 95 °C for 3 min, followed by 36 cycles reaction of 15 s at 96 °C, annealing step at 50 °C for 30 s, elongation for 3 min at 68 °C, and the final elongation step for 5 min at 68 °C. Then products of amplification were collected and mixed for Next Generation Sequencing. Briefly, DNA was fragmented by sonication to produce fragments. A 350 bps fragments library was constructed by NEB Next^®^ Ultra™ DNA Library Prep Kit for Illumina and sequenced on an Illumina NovaSeq 6000 platform (Novogene Bioinformatics Institute, Tianjin, China) using paired-end 150 bps methods. The reads were assembled with SPAdes V.3.14.1 (Bankevich 2012) and MitoZ (Meng 2019). Then the assembled mitogenome was annotated by using the MITOS WebServer (http://mitos2.bioinf.uni-leipzig.de/index.py), and the manual confirmation method with reference to other Curculionidae species.

## Results and discussion

**Figure 1.**
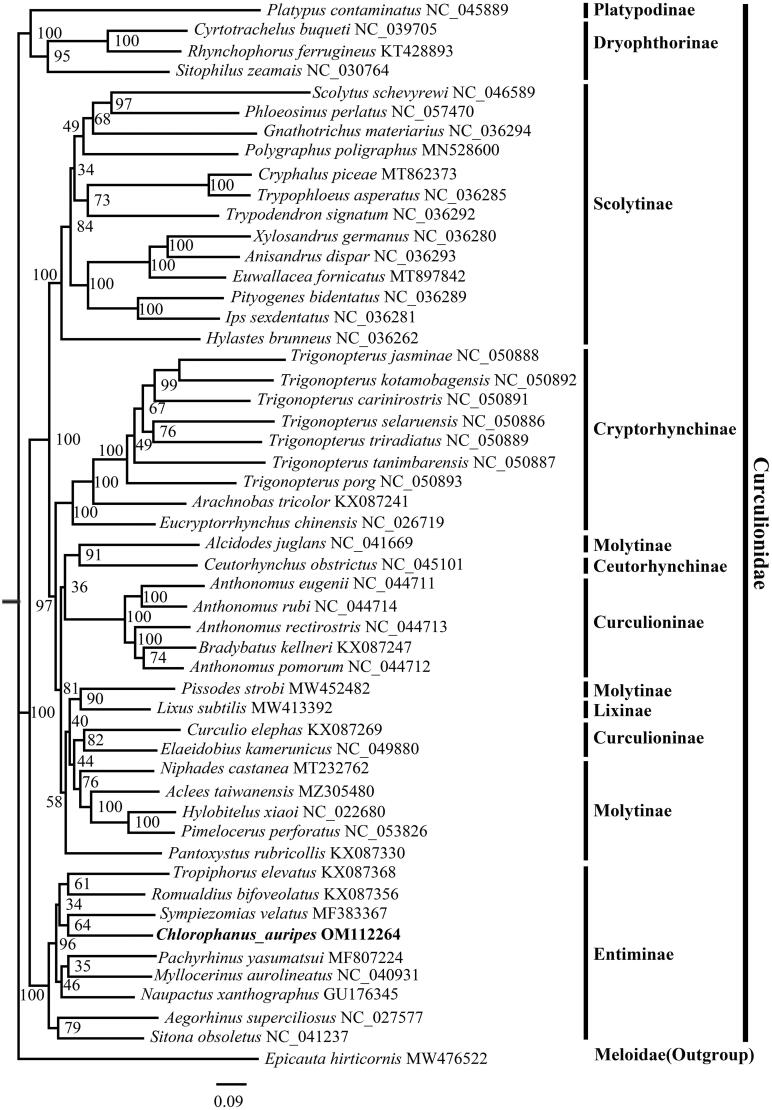
IQ-tree with the maximum likelihood method was constructed using 13 PCG sequence of *C. auripes* with 51 species of Coleoptera. Bootstrap support values in % are shown at nodes. Genbank accession numbers for the sequences are indicated next to the species names. The newly sequenced species are indicated in bold.

The complete mitogenome of *C. auripes* is a circular DNA molecule with a length of 18,149 base pairs and contains 37 genes including 13 protein-coding genes (PCGs), 22 tRNA genes, 2 rRNA genes, and one control region. Its sequence was deposited in GenBank under the accession number OM112264. The phylogenetic relationship was constructed based on the 13 protein-coding genes from 51 species of Curculionidae and 1 outgroup species of Meloidae using the IQ-tree on XSEDE with the maximum-likelihood method (Figure 1). The result highly supported the monophyly of Curculionidae and showed that *C. auripes* was the sister group of *Pachyrhinus yasumatsui*, which is consistent with the result based on 28S rDNA gene, CAD gene, ArgK gene, EF-1α gene, and COI gene (Jordal 2011).

## Ethical approval

This study was approved by the Administration Committee of Experimental Animals, Qinghai Province, China.

## Author contributions

Xiaoning Zhang was involved in the conception and design. Guoxiang Zhang was involved in the PCR experiments. Qingbai Hou was involved in the analysis and interpretation of the data. All authors contributed to the final manuscript and agree to the final approval of the version to be published. All authors agree to be accountable for all aspects of the work.

## Data Availability

The complete mitochondrial genome sequences of *Chlorophanus auripes* have been deposited in GenBank under the accession number OM112264. The associated BioProject and Bio-Sample numbers are PRJNA793188, SAMN24518687, SRA for short reads is SRR17456099.
